# Bis[μ-1,3-bis(4,5-dihydroimidazol-2-yl)benzene-κ^2^ *N*:*N*′]bis[dichloridozinc(II)] *N*,*N*′-dimethylformamide disolvate

**DOI:** 10.1107/S1600536808027773

**Published:** 2008-09-06

**Authors:** Lin Cheng, Ya-Wen Zhang, Yan-Yan Sun, Gong Zhang

**Affiliations:** a Department of Chemistry and Chemical Engineering, Southeast University, Nanjing, People’s Republic of China; b Department of Chemistry and Chemical Engineering, State Key Laboratory of Coordination Chemistry, Nanjing University, Nanjing, People’s Republic of China

## Abstract

The title compound, [Zn_2_Cl_4_(C_12_H_14_N_4_)_2_]·2C_3_H_7_NO, is located on a centre of inversion with one half of a complex mol­ecule and one dimethyl­formamide solvent mol­ecule in the asymmetric unit. The Zn^II^ ion is tetra­hedrally coordinated by two organic ligands and two chloride ions. Each organic ligand acts as a bidentate ligand, connecting two Zn^II^ ions, resulting in a dimeric [2:2] metallamacrocyclic structure. Adjacent mol­ecules are further linked by N—H⋯Cl hydrogen bonds and the solvent is linked to the complex by N—H⋯O hydrogen bonds.

## Related literature

For related structures, see: Ren *et al.* (2004 ▶, 2007 ▶).
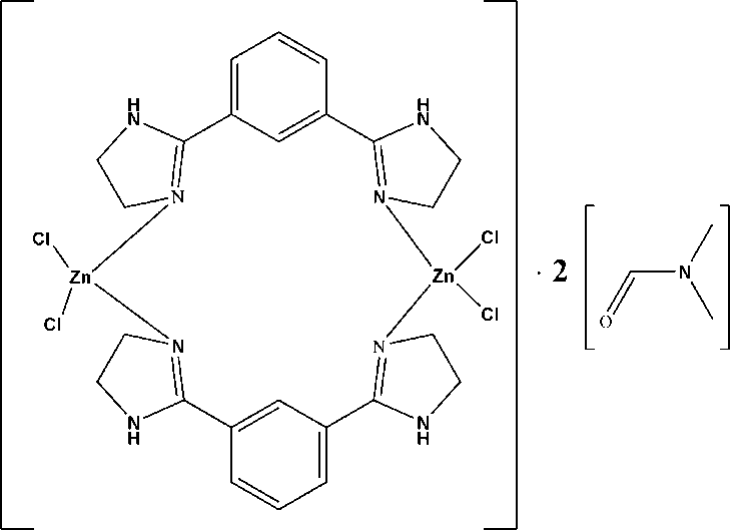


## Experimental

### 

#### Crystal data

[Zn_2_Cl_4_(C_12_H_14_N_4_)_2_]·2C_3_H_7_NO
*M* *_r_* = 847.28
Monoclinic, 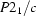

*a* = 8.1774 (11) Å
*b* = 8.5032 (12) Å
*c* = 27.097 (4) Å
β = 92.890 (2)°
*V* = 1881.8 (4) Å^3^
*Z* = 2
Mo *K*α radiation
μ = 1.60 mm^−1^
*T* = 123 (2) K
0.43 × 0.27 × 0.20 mm

#### Data collection

Bruker APEX CCD diffractometer
Absorption correction: multi-scan (*SADABS*; Sheldrick, 2000 ▶) *T*_min_ = 0.546, *T*_max_ = 0.740
13375 measured reflections
3659 independent reflections
3215 reflections with *I* > 2σ(*I*)
*R*_int_ = 0.043

#### Refinement

*R*[*F* ^2^ > 2σ(*F* ^2^)] = 0.034
*wR*(*F* ^2^) = 0.087
*S* = 1.05
3659 reflections
217 parameters
H-atom parameters constrained
Δρ_max_ = 0.54 e Å^−3^
Δρ_min_ = −0.48 e Å^−3^

### 

Data collection: *SMART* (Bruker, 2000 ▶); cell refinement: *SMART*; data reduction: *SAINT* (Bruker, 2000 ▶); program(s) used to solve structure: *SHELXS97* (Sheldrick, 2008 ▶); program(s) used to refine structure: *SHELXL97* (Sheldrick, 2008 ▶); molecular graphics: *SHELXTL* (Sheldrick, 2008 ▶); software used to prepare material for publication: *SHELXTL*.

## Supplementary Material

Crystal structure: contains datablocks I, global. DOI: 10.1107/S1600536808027773/bt2778sup1.cif

Structure factors: contains datablocks I. DOI: 10.1107/S1600536808027773/bt2778Isup2.hkl

Additional supplementary materials:  crystallographic information; 3D view; checkCIF report

## Figures and Tables

**Table 1:** Hydrogen-bond geometry (Å, °)

*D*—H⋯*A*	*D*—H	H⋯*A*	*D*⋯*A*	*D*—H⋯*A*
N1—H1*C*⋯Cl2^i^	0.88	2.74	3.241 (2)	117
N3—H3*A*⋯O1	0.88	2.12	2.870 (3)	143

Symmetry code: (i) 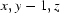.

**Fig. 1.**
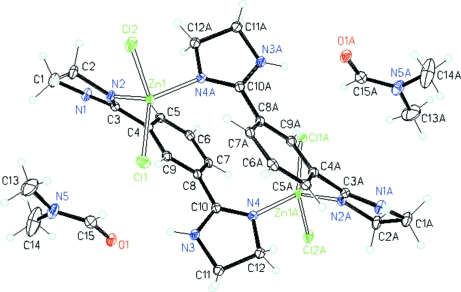
The title compound with 30% thermal ellipsoids. Symmetry code: a: 1 - x, 1 - y, -z.

**Fig. 2.**
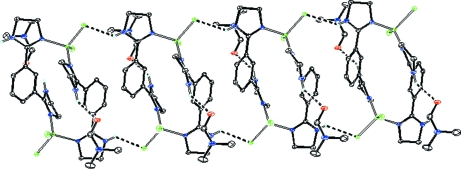
Partial packing diagram of the title compound. The H atoms bonded to C atoms are omitted for clarity.

## supplementary crystallographic information

### Comment

Recently, the photophysical properties of coordination compounds of d_10_ monovalent ions of the coinage metals have been of great interests. And metallomacrocyclic compounds are a rapidly growing field concerning due to their rich luminescent properties (Ren *et al.* 2004). Here, we present the syntheses and structural characterization of a dimeric [2:2] metallomacrocyclic compound [Zn_2_(bib)_2_Cl_2_].2DMF (bib = 1,3-bis(4,5-Dihydro-1*H*-imidazol-2-yl)benzene).

The asymmetric unit of the title compound, [Zn_2_(bib)_2_Cl_2_].2DMF, contains one Zn(II) cation, one bib ligand, two chloride ions and one DMF molecule. In the compound, the Zn(II) ion displays a tetrahedral geometry, being surrounded by two bib ligands and two chloride ions. Each bib acts as a bidentate ligand and every two bib ligands ligate a pair of Zn(II) ions resulting in a dimeric [2:2] metallomacrocyclic structure. Adjacent molecules are further linked   by the N-H···Cl hyrogen bonds and the solvent is linked to the complex by N-H···O hydrogen bonds.

### Experimental

To a solution of ZnCl_2_.2H_2_O (0.172 g, 1 mmol) in CH_3_OH (5 ml), an aqueous solution (5 ml) of bib (0.214 g, 1 mmol) was added. After the mixture was stirred for half an hour, white precipitate was filtrated, dried and collected. Then the white solids were completely dissolved into 2 ml DMF by heating. The DMF solution are placed into a glass test tube, and ether vapors were slowly diffused into the solution. After four weeks, colorless block crystals were obtained [yield 10% (8.5 mg) based on Zn(II)].

### Refinement

All   H atoms were positioned geometrically and refined using a riding model with C—H = 0.95 and 0.99 Å with *U*_iso_(H) = 1.2 *U*_iso_(C), and N —H = 0.88 Å with *U*_iso_(H) = 1.2 *U*_iso_(N).

### Crystal data

**Table:**

[Zn_2_Cl_4_(C_12_H_14_N_4_)_2_]·2C_3_H_7_NO	*F*(000) = 872
*M**_r_* = 847.28	*D*_x_ = 1.495 Mg m^−^^3^
Monoclinic, *P*2_1_/*c*	Mo *K*α radiation, λ = 0.71073 Å
Hall symbol: -P 2ybc	Cell parameters from 781 reflections
*a* = 8.1774 (11) Å	θ = 2.4–28.0°
*b* = 8.5032 (12) Å	µ = 1.60 mm^−^^1^
*c* = 27.097 (4) Å	*T* = 123 K
β = 92.890 (2)°	Block, colorless
*V* = 1881.8 (4) Å^3^	0.43 × 0.27 × 0.20 mm
*Z* = 2	

### Data collection

**Table:**

Bruker APEX CCD diffractometer	3659 independent reflections
Radiation source: fine-focus sealed tube	3215 reflections with *I* > 2σ(*I*)
graphite	*R*_int_ = 0.043
phi and ω scan	θ_max_ = 26.0°, θ_min_ = 2.8°
Absorption correction: multi-scan (SADABS; Sheldrick, 2000)	*h* = −10→9
*T*_min_ = 0.546, *T*_max_ = 0.740	*k* = −10→10
13375 measured reflections	*l* = −31→33

### Refinement

**Table:**

Refinement on *F*^2^	Primary atom site location: structure-invariant direct methods
Least-squares matrix: full	Secondary atom site location: difference Fourier map
*R*[*F*^2^ > 2σ(*F*^2^)] = 0.034	Hydrogen site location: inferred from neighbouring sites
*wR*(*F*^2^) = 0.087	H-atom parameters constrained
*S* = 1.05	*w* = 1/[σ^2^(*F*_o_^2^) + (0.0445*P*)^2^ + 0.1856*P*] where *P* = (*F*_o_^2^ + 2*F*_c_^2^)/3
3659 reflections	(Δ/σ)_max_ = 0.001
217 parameters	Δρ_max_ = 0.54 e Å^−^^3^
0 restraints	Δρ_min_ = −0.48 e Å^−^^3^

### Special details

**Table:**

Geometry. All e.s.d.'s (except the e.s.d. in the dihedral angle between two l.s. planes) are estimated using the full covariance matrix. The cell e.s.d.'s are taken into account individually in the estimation of e.s.d.'s in distances, angles and torsion angles; correlations between e.s.d.'s in cell parameters are only used when they are defined by crystal symmetry. An approximate (isotropic) treatment of cell e.s.d.'s is used for estimating e.s.d.'s involving l.s. planes.
Refinement. Refinement of *F*^2^ against ALL reflections. The weighted *R*-factor *wR* and goodness of fit *S* are based on *F*^2^, conventional *R*-factors *R* are based on *F*, with *F* set to zero for negative *F*^2^. The threshold expression of *F*^2^ > σ(*F*^2^) is used only for calculating *R*-factors(gt) *etc*. and is not relevant to the choice of reflections for refinement. *R*-factors based on *F*^2^ are statistically about twice as large as those based on *F*, and *R*- factors based on ALL data will be even larger.

### Fractional atomic coordinates and isotropic or equivalent isotropic displacement parameters (Å^2^)

**Table:**

	*x*	*y*	*z*	*U*_iso_*/*U*_eq_	
Zn1	0.48008 (3)	0.59217 (3)	0.126447 (9)	0.01901 (11)	
Cl1	0.75119 (7)	0.59787 (7)	0.12124 (2)	0.03078 (16)	
Cl2	0.39514 (8)	0.76920 (7)	0.18274 (2)	0.03058 (17)	
N1	0.3153 (3)	0.1342 (2)	0.15690 (7)	0.0258 (5)	
H1C	0.2681	0.0467	0.1462	0.031*	
N2	0.4140 (2)	0.3782 (2)	0.15104 (7)	0.0207 (4)	
N3	0.8313 (2)	0.3109 (2)	−0.00124 (7)	0.0229 (4)	
H3A	0.8515	0.2981	0.0307	0.028*	
N4	0.6803 (2)	0.3684 (2)	−0.06974 (7)	0.0204 (4)	
C1	0.3390 (4)	0.1829 (3)	0.20863 (9)	0.0317 (6)	
H1A	0.4348	0.1300	0.2251	0.038*	
H1B	0.2406	0.1617	0.2274	0.038*	
C2	0.3685 (3)	0.3594 (3)	0.20290 (9)	0.0287 (6)	
H2A	0.2681	0.4198	0.2091	0.034*	
H2B	0.4581	0.3955	0.2261	0.034*	
C3	0.3786 (3)	0.2473 (3)	0.12837 (8)	0.0196 (5)	
C4	0.3941 (3)	0.2201 (3)	0.07490 (8)	0.0190 (5)	
C5	0.2771 (3)	0.1283 (3)	0.04869 (9)	0.0211 (5)	
H5A	0.1897	0.0817	0.0653	0.025*	
C6	0.2896 (3)	0.1059 (3)	−0.00154 (9)	0.0221 (5)	
H6A	0.2091	0.0454	−0.0195	0.026*	
C7	0.4181 (3)	0.1707 (3)	−0.02578 (8)	0.0200 (5)	
H7A	0.4255	0.1550	−0.0603	0.024*	
C8	0.5371 (3)	0.2590 (2)	0.00036 (8)	0.0185 (5)	
C9	0.5245 (3)	0.2843 (3)	0.05072 (8)	0.0190 (5)	
H9A	0.6048	0.3453	0.0686	0.023*	
C10	0.6822 (3)	0.3157 (3)	−0.02469 (8)	0.0188 (5)	
C11	0.9542 (3)	0.3309 (3)	−0.03839 (8)	0.0248 (5)	
H11A	0.9963	0.2285	−0.0496	0.030*	
H11B	1.0469	0.3971	−0.0259	0.030*	
C12	0.8515 (3)	0.4138 (3)	−0.07927 (9)	0.0256 (5)	
H12A	0.8659	0.5292	−0.0772	0.031*	
H12B	0.8819	0.3774	−0.1123	0.031*	
N5	0.8751 (3)	0.1284 (3)	0.16839 (8)	0.0381 (6)	
C13	0.8524 (5)	0.2201 (5)	0.21257 (12)	0.0666 (11)	
H13A	0.8509	0.3322	0.2042	0.100*	
H13B	0.9426	0.1990	0.2369	0.100*	
H13C	0.7483	0.1911	0.2265	0.100*	
C14	0.8795 (7)	−0.0394 (5)	0.17315 (14)	0.0913 (17)	
H14A	0.8958	−0.0869	0.1408	0.137*	
H14B	0.7758	−0.0766	0.1855	0.137*	
H14C	0.9698	−0.0697	0.1963	0.137*	
C15	0.8906 (3)	0.1948 (3)	0.12484 (10)	0.0328 (6)	
H15A	0.8902	0.3064	0.1239	0.039*	
O1	0.9055 (2)	0.1264 (2)	0.08571 (6)	0.0313 (4)	

### Atomic displacement parameters (Å^2^)

**Table:**

	*U*^11^	*U*^22^	*U*^33^	*U*^12^	*U*^13^	*U*^23^
Zn1	0.02203 (17)	0.02012 (16)	0.01476 (16)	0.00103 (10)	−0.00025 (11)	0.00084 (10)
Cl1	0.0216 (3)	0.0318 (3)	0.0388 (4)	−0.0006 (2)	−0.0008 (3)	0.0019 (3)
Cl2	0.0501 (4)	0.0242 (3)	0.0176 (3)	0.0052 (3)	0.0033 (3)	−0.0022 (2)
N1	0.0358 (12)	0.0240 (10)	0.0179 (10)	−0.0066 (9)	0.0033 (9)	0.0009 (8)
N2	0.0265 (11)	0.0222 (10)	0.0135 (10)	0.0017 (8)	0.0020 (8)	0.0011 (7)
N3	0.0216 (11)	0.0327 (11)	0.0144 (10)	−0.0011 (9)	−0.0005 (8)	0.0020 (8)
N4	0.0194 (10)	0.0252 (10)	0.0167 (10)	0.0023 (8)	0.0026 (8)	0.0006 (8)
C1	0.0473 (17)	0.0289 (13)	0.0194 (13)	−0.0010 (12)	0.0065 (11)	0.0035 (10)
C2	0.0439 (16)	0.0279 (13)	0.0145 (12)	0.0012 (11)	0.0038 (11)	0.0021 (10)
C3	0.0179 (12)	0.0227 (11)	0.0180 (12)	0.0022 (9)	0.0003 (9)	0.0033 (9)
C4	0.0210 (12)	0.0178 (11)	0.0182 (12)	0.0022 (9)	0.0009 (9)	−0.0002 (9)
C5	0.0190 (12)	0.0204 (11)	0.0240 (13)	0.0017 (9)	0.0014 (10)	0.0028 (9)
C6	0.0215 (13)	0.0194 (11)	0.0245 (13)	0.0020 (9)	−0.0065 (10)	−0.0011 (9)
C7	0.0235 (13)	0.0201 (11)	0.0160 (11)	0.0045 (9)	−0.0021 (9)	0.0007 (9)
C8	0.0212 (12)	0.0184 (11)	0.0159 (11)	0.0028 (9)	−0.0006 (9)	0.0015 (9)
C9	0.0210 (12)	0.0186 (11)	0.0171 (12)	0.0018 (9)	−0.0010 (9)	−0.0009 (9)
C10	0.0233 (13)	0.0169 (10)	0.0161 (12)	0.0019 (9)	−0.0002 (9)	−0.0024 (9)
C11	0.0198 (12)	0.0338 (13)	0.0208 (12)	0.0004 (10)	0.0008 (10)	0.0015 (10)
C12	0.0217 (13)	0.0340 (14)	0.0211 (13)	0.0005 (10)	0.0017 (10)	0.0049 (10)
N5	0.0468 (15)	0.0488 (15)	0.0192 (12)	−0.0060 (11)	0.0050 (10)	−0.0011 (10)
C13	0.071 (3)	0.096 (3)	0.0334 (19)	0.009 (2)	0.0134 (17)	−0.0192 (18)
C14	0.184 (5)	0.052 (2)	0.038 (2)	−0.034 (3)	0.008 (3)	0.0071 (18)
C15	0.0330 (16)	0.0336 (14)	0.0318 (15)	0.0057 (12)	0.0013 (11)	0.0033 (12)
O1	0.0307 (10)	0.0468 (11)	0.0163 (9)	0.0015 (8)	−0.0001 (7)	0.0016 (8)

### Geometric parameters (Å, °)

**Table:**

Zn1—N4^i^	1.9978 (19)	C5—H5A	0.9500
Zn1—N2	2.0205 (19)	C6—C7	1.382 (3)
Zn1—Cl1	2.2291 (7)	C6—H6A	0.9500
Zn1—Cl2	2.2764 (7)	C7—C8	1.394 (3)
N1—C3	1.353 (3)	C7—H7A	0.9500
N1—C1	1.465 (3)	C8—C9	1.390 (3)
N1—H1C	0.8800	C8—C10	1.477 (3)
N2—C3	1.298 (3)	C9—H9A	0.9500
N2—C2	1.480 (3)	C11—C12	1.528 (3)
N3—C10	1.347 (3)	C11—H11A	0.9900
N3—C11	1.468 (3)	C11—H11B	0.9900
N3—H3A	0.8800	C12—H12A	0.9900
N4—C10	1.300 (3)	C12—H12B	0.9900
N4—C12	1.487 (3)	N5—C15	1.320 (3)
N4—Zn1^i^	1.9978 (19)	N5—C14	1.433 (4)
C1—C2	1.529 (3)	N5—C13	1.449 (4)
C1—H1A	0.9900	C13—H13A	0.9800
C1—H1B	0.9900	C13—H13B	0.9800
C2—H2A	0.9900	C13—H13C	0.9800
C2—H2B	0.9900	C14—H14A	0.9800
C3—C4	1.479 (3)	C14—H14B	0.9800
C4—C9	1.391 (3)	C14—H14C	0.9800
C4—C5	1.400 (3)	C15—O1	1.221 (3)
C5—C6	1.383 (3)	C15—H15A	0.9500
			
N4^i^—Zn1—N2	103.21 (8)	C6—C7—C8	120.1 (2)
N4^i^—Zn1—Cl1	124.35 (6)	C6—C7—H7A	120.0
N2—Zn1—Cl1	108.90 (6)	C8—C7—H7A	120.0
N4^i^—Zn1—Cl2	101.17 (6)	C9—C8—C7	119.9 (2)
N2—Zn1—Cl2	106.18 (6)	C9—C8—C10	120.1 (2)
Cl1—Zn1—Cl2	111.47 (3)	C7—C8—C10	119.9 (2)
C3—N1—C1	108.03 (19)	C8—C9—C4	119.9 (2)
C3—N1—H1C	126.0	C8—C9—H9A	120.1
C1—N1—H1C	126.0	C4—C9—H9A	120.1
C3—N2—C2	107.20 (19)	N4—C10—N3	114.9 (2)
C3—N2—Zn1	132.35 (16)	N4—C10—C8	124.9 (2)
C2—N2—Zn1	119.66 (14)	N3—C10—C8	120.2 (2)
C10—N3—C11	107.93 (18)	N3—C11—C12	100.39 (18)
C10—N3—H3A	126.0	N3—C11—H11A	111.7
C11—N3—H3A	126.0	C12—C11—H11A	111.7
C10—N4—C12	106.62 (19)	N3—C11—H11B	111.7
C10—N4—Zn1^i^	139.15 (17)	C12—C11—H11B	111.7
C12—N4—Zn1^i^	114.22 (14)	H11A—C11—H11B	109.5
N1—C1—C2	101.20 (19)	N4—C12—C11	104.02 (19)
N1—C1—H1A	111.5	N4—C12—H12A	111.0
C2—C1—H1A	111.5	C11—C12—H12A	111.0
N1—C1—H1B	111.5	N4—C12—H12B	111.0
C2—C1—H1B	111.5	C11—C12—H12B	111.0
H1A—C1—H1B	109.3	H12A—C12—H12B	109.0
N2—C2—C1	104.55 (19)	C15—N5—C14	120.2 (3)
N2—C2—H2A	110.8	C15—N5—C13	122.1 (3)
C1—C2—H2A	110.8	C14—N5—C13	117.7 (3)
N2—C2—H2B	110.8	N5—C13—H13A	109.5
C1—C2—H2B	110.8	N5—C13—H13B	109.5
H2A—C2—H2B	108.9	H13A—C13—H13B	109.5
N2—C3—N1	115.0 (2)	N5—C13—H13C	109.5
N2—C3—C4	124.7 (2)	H13A—C13—H13C	109.5
N1—C3—C4	120.2 (2)	H13B—C13—H13C	109.5
C9—C4—C5	120.0 (2)	N5—C14—H14A	109.5
C9—C4—C3	120.4 (2)	N5—C14—H14B	109.5
C5—C4—C3	119.6 (2)	H14A—C14—H14B	109.5
C6—C5—C4	119.6 (2)	N5—C14—H14C	109.5
C6—C5—H5A	120.2	H14A—C14—H14C	109.5
C4—C5—H5A	120.2	H14B—C14—H14C	109.5
C7—C6—C5	120.5 (2)	O1—C15—N5	126.3 (3)
C7—C6—H6A	119.7	O1—C15—H15A	116.9
C5—C6—H6A	119.7	N5—C15—H15A	116.9

Symmetry codes: (i) −*x*+1, −*y*+1, −*z*.

### Hydrogen-bond geometry (Å, °)

**Table:**

*D*—H···*A*	*D*—H	H···*A*	*D*···*A*	*D*—H···*A*
N1—H1C···Cl2^ii^	0.88	2.74	3.241 (2)	117.
N3—H3A···O1	0.88	2.12	2.870 (3)	143.

Symmetry codes: (ii) *x*, *y*−1, *z*.
